# *Bacillus velezensis* strain Ag75 as a new multifunctional agent for biocontrol, phosphate solubilization and growth promotion in maize and soybean crops

**DOI:** 10.1038/s41598-022-19515-8

**Published:** 2022-09-10

**Authors:** Mirela Mosela, Galdino Andrade, Luana Rainieri Massucato, Suelen Regina de Araújo Almeida, Alison Fernando Nogueira, Renato Barros de Lima Filho, Douglas Mariani Zeffa, Silas Mian, Allan Yukio Higashi, Gabriel Danilo Shimizu, Gustavo Manoel Teixeira, Kelvin Shinohata Branco, Marcos Ventura Faria, Renata Mussoi Giacomin, Carlos Alberto Scapim, Leandro Simões Azeredo Gonçalves

**Affiliations:** 1 Microbiology Department, Universidade Estadual de Londrina (UEL), Londrina, 86051-900 Brazil; 2 Agronomy Department, Universidade Estadual de Londrina (UEL), Londrina, 86051-900 Brazil; 3 Agronomy Department, Universidade Estadual de Maringá (UEM), Maringá, 87020-900 Brazil; 4 Agronomy Department, Universidade Estadual do Centro Oeste (Unicentro), Guarapuava, 85040-167 Brazil; 5 Biology Department, Universidade Estadual do Centro Oeste (Unicentro), Guarapuava, 85040-167 Brazil

**Keywords:** Abiotic, Bacterial development

## Abstract

Soybean and maize are some of the main drivers of Brazilian agribusiness. However, biotic and abiotic factors are of great concern, causing huge grain yield and quality losses. Phosphorus (P) deficiency is important among the abiotic factors because most Brazilian soils have a highly P-fixing nature. Thus, large amounts of phosphate fertilizers are regularly applied to overcome the rapid precipitation of P. Searching for alternatives to improve the use of P by crops is essential to reduce the demand for P input. The use of multifunctional rhizobacteria can be considered one of these alternatives. In this sense, the objective of the present work was to select and validate bacterial strains with triple action (plant growth promoter, phosphate solubilizer, and biocontrol agent) in maize and soybean, aiming to develop a multifunctional microbial inoculant for Brazilian agriculture. Bacterial strains with high indole acetic acid (IAA) production, phosphate solubilization, and antifungal activity against soil pathogenic fungi (*Rhizoctonia solani*, *Macrophomina phaseolina*, and *Fusarium solani*) were selected from the maize rhizosphere. Then, they were evaluated as growth promoters in maize under greenhouse conditions. Based on this study, strain 03 (Ag75) was selected due to its high potential for increasing biomass (root and shoot) and shoot P content in maize. This strain was identified through genomic sequencing as *Bacillus velezensis*. In field experiments, the inoculation of this bacterium increased maize and soybean yields by 17.8 and 26.5%, respectively, compared to the control (25 kg P_2_O_5_). In addition, the inoculation results did not differ from the control with 84 kg P_2_O_5_, indicating that it is possible to reduce the application of phosphate in these crops. Thus, the Ag75 strain has great potential for developing a multifunctional microbial inoculant that combines the ability to solubilize phosphate, promote plant growth, and be a biocontrol agent for several phytopathogenic fungi.

## Introduction

Agribusiness is an essential sector of the Brazilian economy, representing 27.4% of the gross domestic product (GDP) and 40.6% (US$ 9.9 billion) of exports in 2021. Approximately 23% of foreign sales in this segment come from the soy complex (grain, meal, and oil) and 8% from maize^1^. In the 2021/2022 harvest, soybean was cultivated on approximately 40.8 million hectares, with a production of 122.2 million t and an average growth of 5.7 million t year^−1^ during the last 10 years. In turn, maize was cultivated on approximately 21.2 million hectares, had a production of 115 million tons, and had an average growth of 3.2 million t year^−1^^2^.

Despite the positive scenario of soybean and maize in Brazil, biotic and abiotic factors generate great concerns, causing huge losses in grain yield and quality. Among the abiotic factors, nutritional deficiency is an important stressor because most tropical soils have high acidity, toxic levels of aluminum (Al), and low nutrient availability, especially phosphorus (P) and nitrogen (N)^3,4^. In the case of P, most Brazilian soils are highly P-fixing soils, and large amounts of phosphate fertilizers are regularly applied to overcome the rapid precipitation of P by iron (Fe^3+^) and Al^3+^ ions^3,5^.

Approximately 60% of the inorganic P fertilizer used in Brazilian agriculture is currently imported, generating a high and unfavorable dependence considering geopolitical fluctuations and dollar volatility^6^. In this context, agricultural management strategies to improve P use efficiency by crops are essential to substantially reduce the demand for P input. Some strategies include increasing soil pH by liming, crop rotation, double cropping, cover crops between seasons, no-tillage, and the use of modern fertilizers. Other approaches involve developing P-use efficient cultivars and inoculating phosphate solubilizing microorganisms (PSM)^5^.

PSM can make P available to plants through several mechanisms, some more related to enzymatic processes (phytases and/or phosphatases) and others to cell physiology, with the extrusion of H^+^ ions and release of organic acids from microbial metabolism^7–9^. Furthermore, some of these microorganisms may have an effect as plant growth promoters and biocontrol agents against plant pathogens^10–12^.

Several strains of bacteria, actinobacteria, and fungi have been reported and investigated for their ability to solubilize phosphate. Among phosphate-solubilizing fungi (PSF), the genera *Aspergillus* and *Penicillium* are the most studied, while phosphate-solubilizing bacteria (PSB) include the genera *Bacillus*, *Pseudomonas*, and *Enterobacter*^7,12^. Some *Bacillus* strains are known to act as plant growth-promoting rhizobacteria (PGPR) either through the solubilization of minerals such as phosphorus or the production of metabolites such as siderophores and phytohormones^13^. In addition, this genus contains excellent root colonizers, having members in the rhizosphere of a wide range of crops and can survive under many stress conditions and control various plant pathogens^13,14^.

The genus *Bacillus* has different tools for controlling phytopathogens, including competition with pathogens for ecological niches and nutrients, production of antimicrobial metabolites, and induction of resistance in the host plant^15,16^. Among the antimicrobial metabolites, *Bacillus* sp. can produce a wide range of antagonist compounds with different structures, having 5–8% of the genome dedicated to the biosynthesis of these secondary metabolites^15^. Non-ribosomal peptides and lipopeptides, polyketide compounds, bacteriocins, and siderophores are the main bioactive molecules controlling plant diseases^16,17^.

The present work aimed to select and validate bacterial strains with triple action (plant growth promoter, phosphate solubilizer, and biocontrol agent) in maize and soybean. The study has the final objective of developing a multifunctional microbial inoculant for Brazilian agriculture, as well as shedding light on the genomic mechanisms associated with these beneficial traits.

## Results

### Greenhouse experiment

By the analysis of variance, a significant effect (p < 0.01) was observed for all the evaluated traits, indicating a wide variability between treatments (Table S3). For stem diameter (SD), the highest values were observed for the control, biomaphos, strain 02, strain 03, strain 04, strain 11, and strain 12 treatments, while for plant height (PH), the highest values were found in strain 02, strain 03, strain 04, strain 10, strain 11, strain 12, and strain 13 (Table S4). For root and shoot dry mass (RDM and SDM), seven and six strains, respectively, obtained higher values than the control, especially strains 03 and 04. For SPC, the treatments that stood out were strain 03, strain 04, strain 05, strain 07, strain 08, and strain 13.

**Figure 1:**
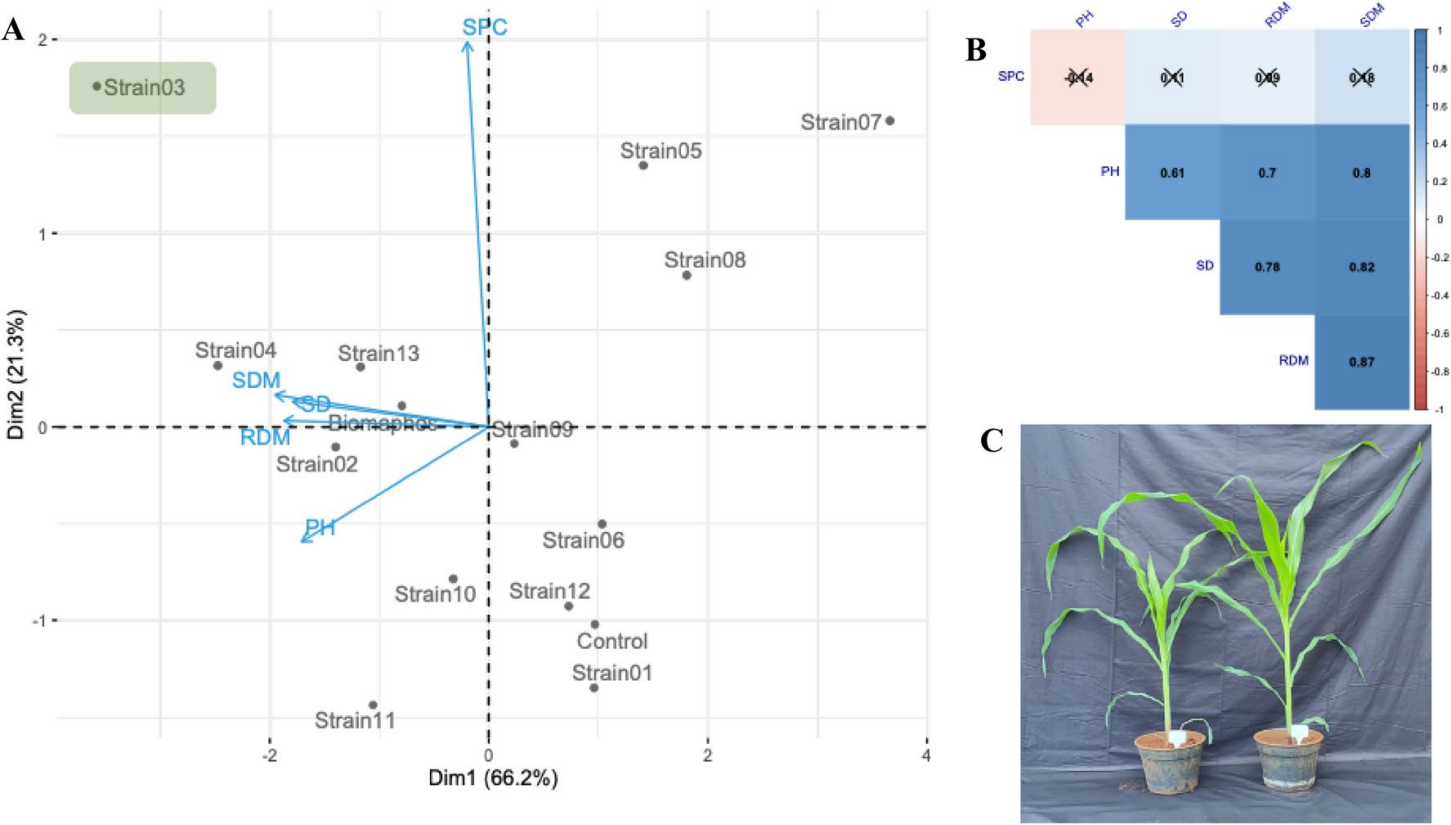
Principal component analysis (**A**) and Pearson correlation (**B**) between traits evaluated in the greenhouse experiment with maize seeds inoculated with different phosphate-solubilizing bacteria. Comparison of the control treatment (without inoculation) with strain 03. *SD* stem diameter, *PH* plant height, *RDM* root dry mass, *SDM* shoot dry mass, *SPC* shoot phosphorus content.

The principal component analysis showed that the first two components explained 87.5% of the total variation (PCA1 and PCA2 with 66.2 and 21.3%, respectively) (Fig. 1A). PH, SD, RDM, and SDM had a high correlation with each other and did not correlate with SPC (Fig. 1B). Strain 03 stood out for all traits, with increments of 16, 45, 42, and 35% for PH, RDM, SDM, and SPC, respectively, in relation to the control (Fig. 1C). Based on these results, strain 03, which was called Ag75, was selected for further experiments.

### Field experiments

Based on the analysis of variance, a significant effect of the environment and treatments (p < 0.01) was observed for grain yield in the maize and soybean experiments (Table 1). For both experiments, no significant effect of the treatment × environment interaction was observed. The coefficients of variation were 10.77 and 11.12 for the maize and soybean experiments, respectively, indicating good experimental precision.

**Table 1:** Analysis of variance for grain yield in maize and soybean experiments with seeds inoculated with phosphate-solubilizing bacteria.

Source of variation^1/^	DF	Maize	DF	Soybean
		Mean square		Mean square
rep/env	18	435,164.4	16	36,016.4
Environment (Env.)	5	76,681,001.6**	4	9,173,354.7**
Treatments (T)	4	6,525,342.7**	4	1,498,355.2**
Env. × T	20	529,651.1^ns^	16	197,868.4^ns^
error	74	617,919.8	60	127,521.2
CV (%)		10.77		11.12
Env.1		8939.23		2519.91
Env.2		7024.80		2515.99
Env.3		5497.00		4060.03
Env.4		4996.57		–
Env.5		10,101.20		3553.94
Env.6		7236.21		3399.64

^1/^Env1.: Londrina (2020/2021), Env2.: Maringá (2020/2021), Env3.: Guarapuava (2020/2021), Env4.: Londrina (2021/2021), Env5.: Londrina (2021/2022) and Env6.: Guarapuava (2021/2022).

^*ns*^, ** and * indicates non-significance, significance at levels 1 and 5% of probability by the F test, respectively.

**Figure 2:**
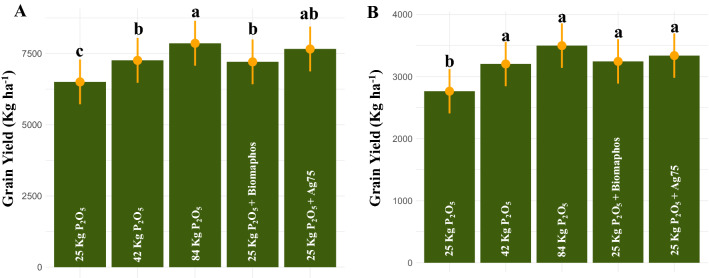
Effect of phosphate solubilizing bacteria on grain yield in maize (**A**) and soybean (**B**) experiments.

For the maize experiments, the average grain yield between environments ranged from 4996.57 (Londrina—2021/2021 off-season) to 10,101.20 kg ha^−1^ (Londrina—2021/2022 harvest) (Table S5). The control treatment—84 kg P_2_O_5_ had the highest average yield (7861.04 kg ha^−1^), not differing statistically from the Ag75 (7660.90 kg ha^−1^) and control—42 kg P_2_O_5_ (7260.58 kg ha^−1^) treatments (Fig. 2A). By unfolding the environments, the biological treatments (Biomaphos and Ag75) did not differ from the control—84 kg P_2_O_5_ in the evaluated environments, indicating the possibility of reducing the application of phosphate in maize. The inoculation of Ag75 allowed an average increase of 17.8% in grain yield in relation to the control—25 kg P_2_O_5_.

For soybean, the average grain yield between environments ranged from 2519.91 (Londrina—2020/2021) to 4060.03 kg ha^−1^ (Guarapuava—2020/2021) (Table S6). The control treatment—25 kg P_2_O_5_ had the lowest productivity, and the Biomaphos, Ag75, control—42 kg P_2_O_5_, and control—84 kg P_2_O_5_ treatments did not differ from each other (Fig. 2B). These treatments obtained an average increase in grain yield of 15.8, 26.5, 17.4, and 20.8%, respectively, in relation to the control—25 kg P_2_O_5_.

For the phosphorus use efficiency indices, significant effects were observed for the environment (p < 0.01) for all variables, while for treatments, only PUtE_g was not significant (Table 2). For treatment × environment interactions, no significant effect was observed. The CV (%) ranged from 21.52 (PUpE_g) to 32.09 (PUE_g—maize). For PUpE_g, the highest values were observed for the biological treatments (Biomaphos and AgPhos), with increases of 23 and 42%, respectively, in relation to the control—25 kg P_2_O_5_. For PUsE_g, in the maize experiment, the highest values were observed for Ag75, Biomaphos, and the control—25 kg P_2_O_5_. For soybean, the highest PUsE values were found in Ag75 and Biomaphos, with increases of 19 and 29%, respectively.

**Table 2:** Analysis of variance and Tukey’s test for phosphorus uptake efficiency (PUpE_g), phosphorus utilization efficiency (PUtE_g), and phosphorus use efficiency (PUsE_g) in maize and soybean experiments with seeds inoculated with phosphate-solubilizing bacteria.

Source of variation^1/^	DF	Maize – mean square			Soybean—mean square
		PUpE_g	PUtE_g	PUsE_g	PUsE_g
rep/env	9	0.02	65,497.1	80,434.7	4206.8
Environment (Env.)	2	0.82**	10,236,391.6**	1,152,283.1**	31,648.3**
Treatments (T)	4	0.67**	81,158.5^ns^	305,295.4**	52,608.2**
Env. × T	8	0.08^ns^	186,044.6^ns^	15,301.0^ns^	1512.5^ns^
Error	36	0.04	129,875.7	54,256.5	1822.7
CV (%)		21.57	26.56	32.09	29.30
**Means**					
25 kg P_2_O_5_		0.66bc	996.4a	524.5ab	165.1b
42 kg P_2_O_5_		0.56cd	970.2a	458.7b	95.8c
84 kg P_2_O_5_		0.34d	1107.6a	332.1b	58.4d
25 kg P_2_O_5_ + Biomaphos		0.83ab	877.2a	681.1a	196.6ab
25 kg P_2_O_5_ + Ag75		0.95a	976.8a	718.4a	212.5a

*ns*, ** and * indicates non-significance, significance at levels 1 and 5% of probability by the F test, respectively.

Means followed by different letters on the same line differ significantly from each other as measured by the Tukey test at the significance level of 5%.

### Antifungal activity

**Figure 3:**
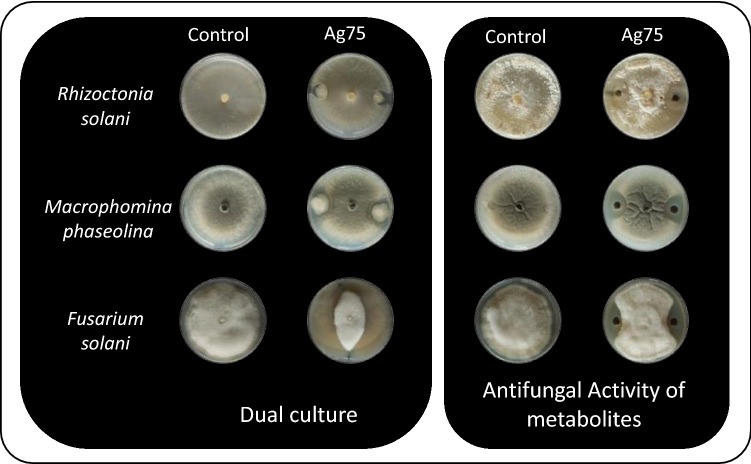
Mycelial growth inhibition of the fungi *Rhizoctonia solani*, *Macrophomina phaseolina*, and *Fusarium solani* using dual culture and cell-free supernatant of the Ag75 strain.

The Ag75 strain showed antifungal activity against *Rhizoctonia solani*, *Macrophomina phaseolina*, and *Fusarium solani*, with percent mycelial growth inhibition of 44, 49 and 61%, respectively, indicating antagonism of this strain against these fungi (Fig. 3). When analyzing the CFS against these fungi, mycelial growth inhibition percentages of 54 and 46% were observed for *M. phaseolina* and *F. solani,* respectively. For *R. solani*, inhibition with CFS was not observed.

### Assembly and annotation of the Ag 75 genome

The CLC Genomics Workbench 11 and IDBA Hybrid genome assembly strategies demonstrated the best results for assembly. A BLASTN search was performed using the largest contig to find a reference genome for the CONTIGuator step. The strain *Bacillus velezensis* NKG-1 was selected to align the contigs and generate pseudocontigs (scaffold). The scaffold contained 22 gaps that were first treated with GapCloser and then manually aligned using Bowtie2 and CLC Genomics Workbench 11. The genome of the Ag75 strain showed a 98.14% alignment rate of reads with a 3,980,135 bp size and a G+C average content of 46.5% (GenBank accession number CP099465; culture collection: CCT8089). A total of 4053 protein-coding genes, 24 rRNA genes, and 85 tRNA genes were found (Figure S1). Most of these genes are associated with functions such as amino acid transport and metabolism, carbohydrate transport and metabolism, transcription, inorganic ion transport and metabolism, and secondary metabolite biosynthesis, transport, and catabolism.

**Figure 4:**
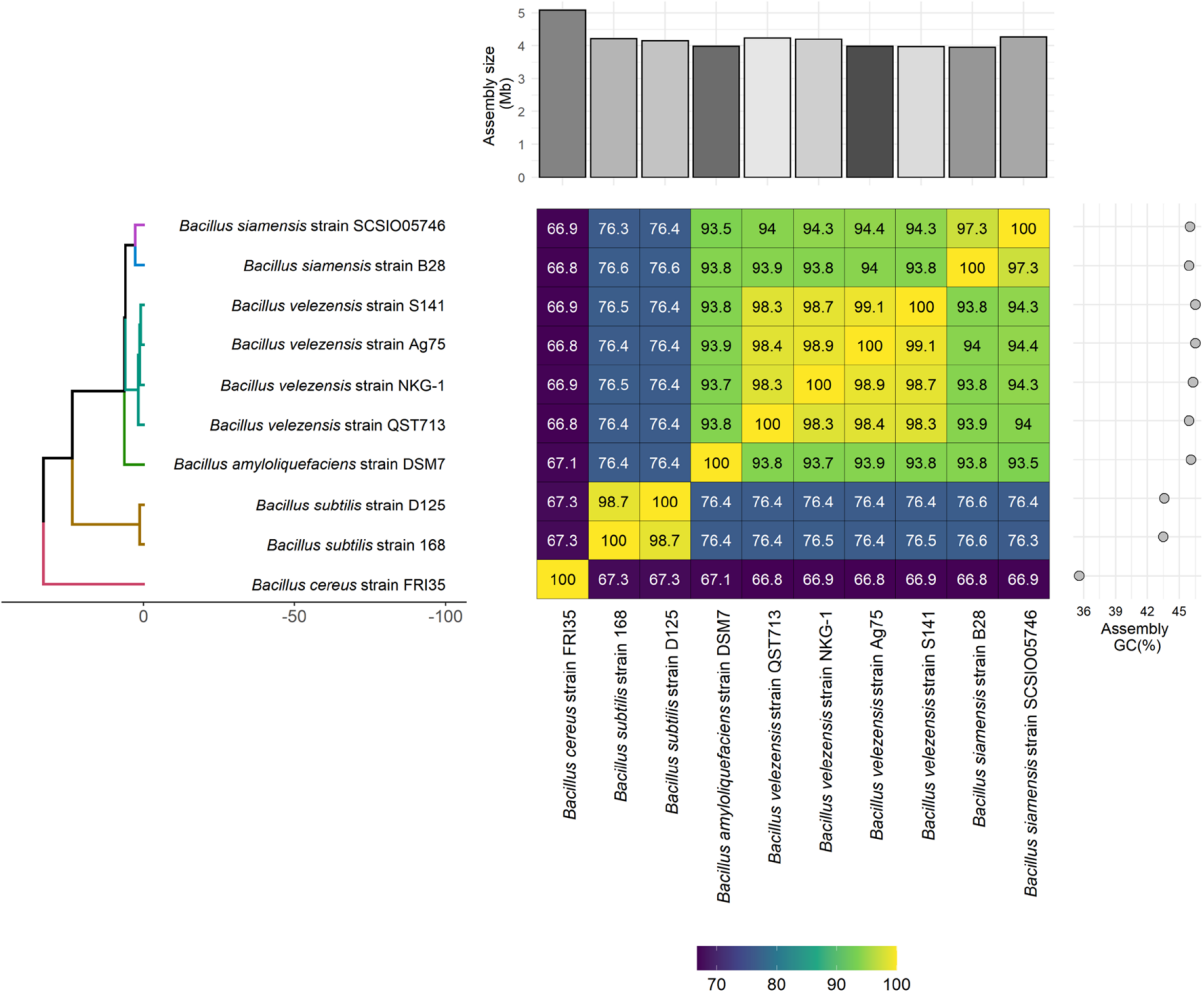
Phylogenetic tree based on maximum likelihood analysis using 10 available genomic assemblies of *Bacillus velezensis*, *Bacillus siamensis*, *Bacillus amyloliquefaciens*, *Bacillus subtilis*, and *Bacillus cereus* with heatmap annotation. The mean nucleotide identity (ANI) values (%) are displayed on the heatmap, ranging from lowest (violet) to highest sequence identity (green–yellow), grouped according to the phylogenetic tree. The heatmap was annotated with a bar graph showing the varying sizes (Mb) of all 10 assemblies (on the top) and their respective GC content (%) (right side).

**Figure 5:**
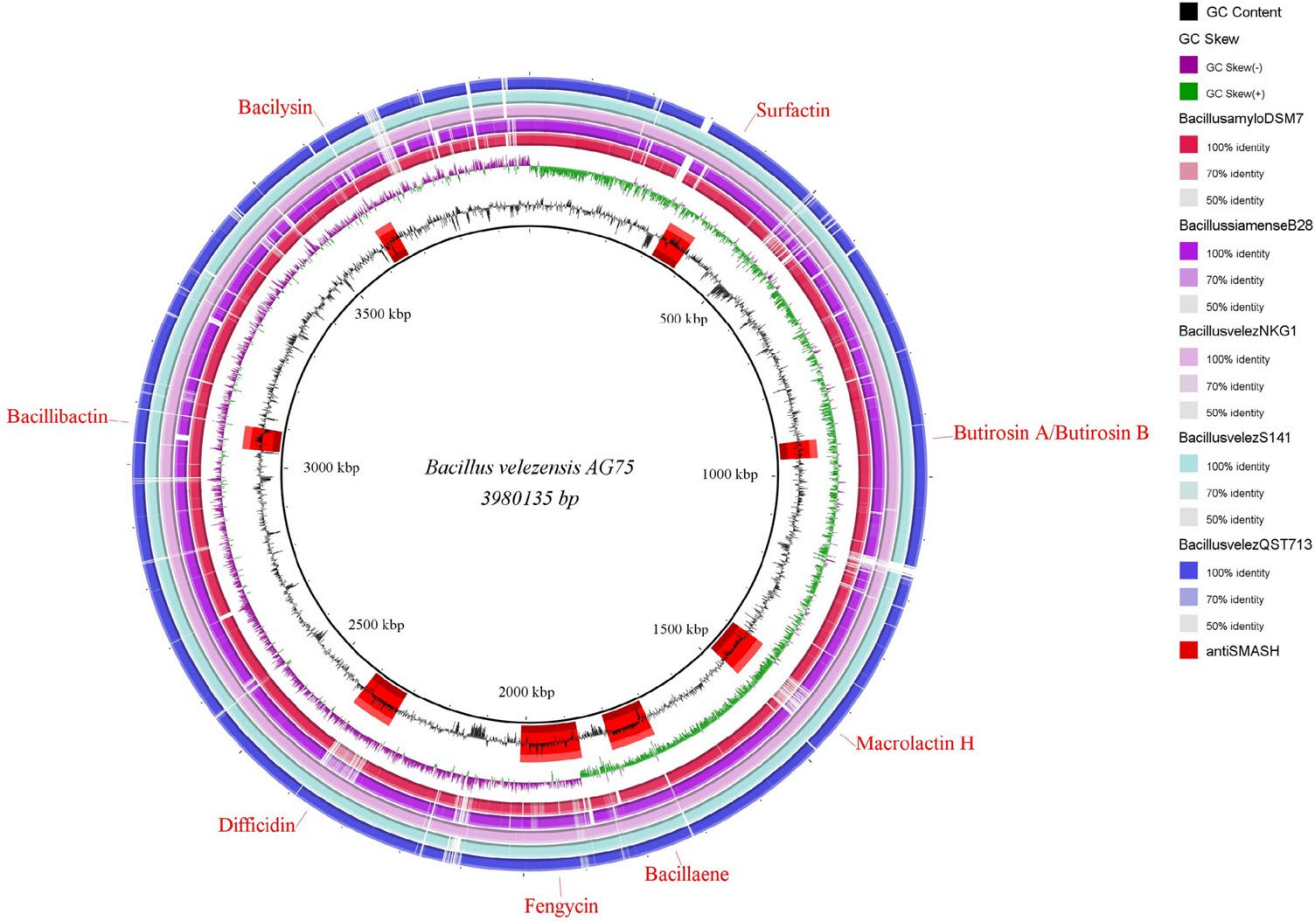
Circular representation of the genome of *Bacillus velezensis* strain Ag75 using the BRIG program. From inside to outside, the legends are as follows: GC content, GC slope, and position of BGCs in the genome indicated by antiSMASH for DSM7, B28, NKG-1, S141, and QST 713.

Comparing the Ag75 strain with the main species of the *Bacillus subtilis* group, the ANI and digital DNA–DNA hybridization (dDDH) were higher with the isolates of *Bacillus velezensis*, with values ranging from 98.44 to 99.12% for ANI and 92.3 to 94.2% for dDDH. In the comparison performed with Gegenees and ortthoANI/GGDC, it was observed that the Ag75 strain is located within the cluster containing most species of *Bacillus velezensis* (Fig. 4). The circular genome of the Ag75 strain is represented in Fig. 5.

### AntiSMASH analysis of secondary metabolites

Using the antiSMASH 5.1.0 webserver, 12 clusters of BGCs were identified in the genome of Ag75 (Table S7). Of these, six clusters showed 100% similarity and were linked to the synthesis of macrolactin, bacillaene, fengycin, difficidin, bacillibactin, and bacilisyn. One cluster had 82% similarity, responsible for surfactin, and another had 7% similarity, responsible for butyrosine A and B synthesis. Four clusters showed no similarity with the database.

### Genetic basis for plant growth-promoting to activities

A number of genes/gene groups associated with plant growth promotion were identified in the Ag75 genome, including production of volatile compounds, phytohormone promoters and phosphatases related to P solubilization (Tables 3, 4). They include 10 putative genes involved in the production of indole-3-acetic acid. In addition, putative genes encoded cytochrome P450 synthase and spermidine acetyltransferase, which are predicted to produce spermidine and polyamine. Other genes encoding proteins involved in the production glucose dehydrogenase, phenazine, trehalose, heat and cold shock, glycine-betaine, and peroxidases, were present. The Ag75 genome has 10 genes involved in the process of biofilm development and regulation and 11 phosphatase genes involved in phosphate solubilization.

**Table 3:** Genes detected in *Bacillus velezensis* Ag75 genome predicated to be involved in plant growth-promoting activity.

Gene ID	Gene name	Protein coded by the gene
**Genes detected in *Bacillus velezensis* Ag75 genome predicated to involved in the production of indole acetic acid (IAA)**		
AG75_002206	*trpA*	Tryptophan synthase subunit alpha
AG75_002207	*trpB*	Tryptophan synthase subunit beta
AG75_002209	*trpC*	Indole-3-glycerol phosphate synthase TrpC
AG75_002210	*trpD*	Anthranilate phosphoribosyltransferase
AG75_002211	*trpE*	Anthranilate synthase component I
AG75_002208	*trpF*	Phosphoribosylanthranilate isomerase
AG75_002829	*ywdH*	Aldehyde dehydrogenase
AG75_000692	*ysnE*	GNAT family *N*-acetyltransferase
AG75_003625	*ywkB*	Auxin efflux carrier family protein
AG75_000284	*amhX*	Amidohydrolase
**Genes detected in *Bacillus velezensis* Ag75 genome predicated to involved in the production spermidine and polyamine**		
AG75_001904	*bioI*	Biotin biosynthesis cytochrome P450
AG75_003671	*speE*	Spermidine synthase
AG75_000568	*bltD*	Spermidine acetyltransferas
**Genes detected in *Bacillus velezensis* Ag75 genome predicated to involved in the production volatile compound (VOC)**		
AG75_003512	*budA*	Acetolactate decarboxylase
AG75_003513	*AlsS*	Acetolactate synthase
AG75_003514	*alsR*	Transcriptional regulator
AG75_00627	*bdhA*	2,3-Butanediol dehydrogenase
AG75_00798	*acoA*	Acetoin dehydrogenase
**Genes detected in *Bacillus velezensis* Ag75 genome predicated to involved in biofilm formation, development, and regulation**		
AG75_001543	*ylbF*	Controls biofilm development
AG75_001749	*ymcA*	Biofilm development
AG75_003250	*ioIW*	Scyllo-inositol 2-dehydrogenase (NADP(1) involved in biofilm formation protein
AG75_001694	*sigD*	RNA polymerase sigma factor for flagellar operon and Biofilm formation
AG75_002430	*sinR*	Master regulator of biofilm formation
AG75_002934	*luxS*	*S*-Ribosyl homocysteine lyase for Quorum sensing Biofilm formation
AG75_003332	*rpoN*	RNA polymerase sigma-54 factor for Biofilm formation
AG75_003444	*csrA*	Carbon storage regulator for Biofilm formation
AG75_003450	*flgM*	Negative regulator of flagellin synthesis f*lgmfor* Biofilm formation
AG75_003473	*wecB*	UDP-N-acetylglucosamine 2-epimerase for Biofilm formation
**Genes detected in *Bacillus velezensis* Ag75 genome predicated to involved to Glucose dehydrogenase**		
AG75_000268	*ycdF*	Glucose 1-dehydrogenase
AG75_000391	*gdh*	Glucose 1-dehydrogenase
AG75_002348	*zwf*	Glucose-6-phosphate dehydrogenase
AG75_003465	*tuaD*	UDP-glucose 6-dehydrogenase
**Genes detected in *Bacillus velezensis* Ag75 genome predicated to involved in Phenazine production and Trehalose metabolism**		
AG75_000825	*phzF*	PhzF family phenazine biosynthesis isomerase
AG75_000762	*treP*	PTS system trehalose-specific EIIBC component
AG75_000764	*treR*	Trehalose operon repressor
**Genes detected in *Bacillus velezensis* Ag75 genome predicated to involved in heat and cold shock**		
AG75_000061	*hslR*	Ribosomal RNA binding protein involved in 50S recycling heat shock protein
AG75_000609	*GroeL*	Heat shock protein 60 kDa family chaperone GroEL
AG75_000608	*GroeS*	Heat shock protein 10 kDa family chaperone GroES
AG75_000504	*cspC*	Cold shock protein CspC
AG75_000897	*cspB*	Cold shock-like protein CspB
AG75_002124	*cspD*	Cold-shock protein CspD
**Genes detected in *Bacillus velezensis* Ag75 genome predicated to involved in Glycine-betaine production**		
AG75_000281	*opuAA*	Glycine/proline betaine ABC transporter ATP-binding protein OpuAA
AG75_000282	*opuAB*	Glycine/proline betaine ABC transporter permease subunit OpuAB
AG75_000283	*opuAC*	Glycine/betaine ABC transporter
AG75_002884	*opuD*	Glycine betaine transporter OpuD
**Genes detected in *Bacillus velezensis* Ag75 genome predicated to involved to Peroxidases**		
AG75_002120	*bsaA*	Glutathione peroxidase
AG75_000854	*bcp*	Thiol peroxidase

**Table 4:** Phosphatase genes detected in *Bacillus velezensis* Ag75 genome predicated to be involved in phosphorus solubilization.

Gene ID	Gene name	Protein coded by the gene
AG75_000252	*phoD*	Phosphodiesterase/alkaline phosphatase D
AG75_000402	*ycsE*	Phosphatase YcsE
AG75_000476	*RsbU*	Serine phosphatase RsbU, regulator of sigma subunit
AG75_000480	*rsbX*	Phosphoserine phosphatase RsbX
AG75_000776	*NG74_RS03905*	Low molecular weight protein tyrosine phosphatase
AG75_001088	*yitU*	Putative phosphatase YitU
AG75_000928	*PhoA*	Alkaline phosphatase
AG75_001511	*suhB*	Inositol-1-monophosphatase
AG75_001622	*prpC*	Protein serine/threonine phosphatase PrpC, regulation of stationary phase
AG75_002780	*phoP*	Alkaline phosphatase synthesis transcriptional regulatory protein PhoP
AG75_002053	*phyC*	3-Phytase

## Discussion

The world faces a major challenge in developing sustainable and eco-friendly methods to improve agricultural productivity. In this sense, great efforts have been made to discover new PGPR to generate microbial inoculants for agriculture. PGPR have multiple mechanisms of action, including increasing soil nutrient availability, regulating microbial communities in the rhizosphere, increasing the proportion of beneficial microorganisms for plants, and producing phytohormones and volatile compounds that promote root growth. Moreover, they act by increasing nutrient absorption, controlling phytopathogens through the production of antimicrobial metabolites and volatile compounds, triggering an induced systemic response in plants, and inducing tolerance to abiotic stresses in plants^15,17,18^. In this context, the search for multifunctional strains with multiple effects of growth promotion, phosphate solubilization, and biocontrol has become essential to developing more effective inoculants for plant development. To this end, we selected multifunctional bacteria from the maize rhizosphere and inferred the potential for application of these strains to obtain a biological product capable of increasing P uptake efficiency, promoting the growth of maize and soybean plants, and acting as a biocontrol agent for the main soil phytopathogenic fungi.

For that, strains of *Bacillus* sp. from the bank of microorganisms with high IAA production, phosphate solubilization, and antifungal activity were selected. IAA plays an important role in root development, mainly in root hair and lateral root formation, improving water and nutrient absorption^19^. In this context, strains with phosphate solubilizing capacity and with high IAA production may increase the area of ​​nutrient uptake and thus enhance the strain's performance in phosphate solubilization. Kudoyarova et al. found that the *Paenibacillus illinoisensis* IB 1087 and *Pseudomonas extremeustralis* IB-ki-13-1A strains selected based on IAA production and phosphate solubilization contributed positively to the development of the wheat root system, favoring greater accumulation of plant biomass and phosphorus^20^. In the present study, seven of the 13 strains evaluated in the greenhouse increased the root system compared to the control. However, no correlation was observed with shoot P content, indicating the complexity of these bacterial × host × environment interaction mechanisms.

Based on the greenhouse study, strain 03 (Ag75) showed a high potential for increasing (root and shoot) biomass and shoot P content in maize. Furthermore, this strain showed antifungal activity against the main soil fungi (*R. solani*, *M. phaseolina*, and *F. solani*), indicating that it is a promising strain for the development of a multifunctional microbial inoculant. By genomic analysis, Ag75 was identified as *Bacillus velezensis*. This species is frequently isolated from different niches (soil, water, rhizosphere, fermented foods, among others), being considered a species adapted to the host and of high economic importance due to its ability to promote plant growth and biocontrol in several economically important crops^21–24^. For example, the FZB42 isolate has already been published in over 140 articles and is related to growth promotion and the identification of antimicrobial compounds responsible for biocontrol. This information is deposited in the ‘AmyloWiki’ database (http://amylowiki.top/)^25^.

For strain Ag75, cyclic lipopeptides (surfactin and fengycin) were identified, which have an important antagonistic effect on several fungal and bacterial pathogens, stimulating plant defense mechanisms and biofilm formation—a key factor for successful colonization of biological control agents^17,26,27^. Another large class of non-ribosomal peptides identified were polyketides (difficidin, bacillaene, and macrolactin), which also play a role in antimicrobial activity^15,17,28^.

In Ag75, the metabolite bacillibactin, an important siderophore, was also identified^29^. This siderophore is highly conserved in the *B. subtilis* group and is induced in response to iron limitation in the environment. It allows *Bacillus* to acquire Fe^3^^+^ and other metals efficiently, thus depriving plant pathogens of this essential element^30,31^. Four of the 12 clusters identified in this strain did not show similarity in the database, and therefore, their products have not yet been identified and described, opening opportunities for further studies.

In addition to producing antimicrobial metabolites, the genome of Ag75 has genes related to plant growth promotion and phosphate solubilization activity. For instance, several identified genes are functionally linked to auxin synthesis and play important roles in the strain’s ability to stimulate plant development^24,32^. In addition, the identified genes related to spermidine and polyamine production are suggested to participate in plant development and growth promotion, involving the production of active metabolites such as steroids, vitamin D3, cholesterol, cytokinin, statins, and terpenes^24^. Ag75 has several phosphatase genes related to phosphorus solubilization, including phytase, which is a particular class of phosphatases capable of mineralizing organic P from phytate and related P organic sources^8,33^. Other studies also verified the presence of phosphatase genes in strains of *Bacillus velezensis*^24,34,35^.

These mechanisms of P solubilization and mineralization combined with those related to promoting root system development favored an increase in P uptake and P use efficiency in maize and soybean when compared to the control without inoculation. Furthermore, these effects were reflected in higher yield increases (17.8% for maize and 26.5% for soybean) in relation to the control 25 kg P_2_O_5_ and did not differ from the control 84 kg P_2_O_5._ Therefore, these results indicate the possibility of reducing phosphate application in these crops. For phosphate solubilization, the Brazilian Agricultural Research Corporation (Embrapa) in partnership with the company Bioma developed an inoculant (Biomaphos) for this purpose. This product is composed of two strains (*B. megaterium* CNPMS B119 and *B. subtilis* CNPMS B2084). Based on the studies by Paiva et al. this inoculant increased maize yield by 8.9%, which is corroborated by the present study with an average increase of 10.8% ^36^.

Brazil has a long history of research on rhizobia and PGPR in various crops. Currently, the use of these products by farmers is a reality, allowing an optimization of mineral fertilizers and contributing to sustainable and low-cost agriculture^37^. Thus, the Ag75 strain has great potential for the development of a multifunctional microbial inoculant that combines the ability to solubilize phosphate, promote plant growth, and act as a biocontrol agent for several phytopathogenic fungi.

## Materials and methods

### Bacterial strain

Thirteen strains of *Bacillus* sp. from the microorganism bank of the AgBio company were used for this study. These strains were isolated from maize rhizospheric soil collected in the municipality of Italva, Rio de Janeiro, Brazil, and selected based on the screening performed for indole acetic acid (IAA) production, phosphate solubilization, and antifungal activity against soil pathogenic fungi (*Rhizoctonia solani*, *Macrophomina phaseolina*, and *Fusarium solani*).

### Bacterial strain preparation

Strains stored at − 80 °C in cryovials containing liquid TSB and glycerol in a 2:1 ratio were activated in Petri dishes containing LBA (Luria Bertani Agar, Neogen Corporation, United States) culture medium at 28 °C for 24 h. A pre-inoculum of each strain was prepared from pure colonies suspended in saline solution (0.85% sodium chloride). The turbidity was adjusted to 0.5 standard on the McFarland nephelometric scale (1.5 × 10^8^ CFU mL^−1^). Thirty microliters of these bacterial suspensions were transferred to 125 mL Erlenmeyer flasks containing 30 mL of AgO2 culture medium (g L^−1^: glucose 15.0, sucrose 10.0, yeast extract 10.0, micronized soy protein 10.0, KH_2_PO_4_ 1.5, MgSO_4_ 0.5, MnSO_4_ 0.5, and CaCl_2_ 1.5, pH 8.0) and incubated at 30 °C for 18–20 h at 200 rpm in an orbital shaker incubator (Orbital shaker Nova Tecnica—NT 735, Brazil) for inoculum production. For fermentation, 1 L Erlenmeyer flasks containing 400 mL of AgO2 culture medium were inoculated with a 4 mL aliquot of the inoculum and incubated at 30 °C for 72 h at 200 rpm. After fermentation, the production concentration was adjusted to 2.0 × 10^9^ CFU mL^−1^.

### Greenhouse experiment

Maize seeds of the cultivar P3340VYHR (Corteva^®^) were inoculated with the bacterial strains at 2 × 10^9^ CFU mL^−1^, constituting 13 treatments. Biomaphos^®^ (*Bacillus subtilis* strain CNPMS B2084 and *Bacillus megaterium* strain CNPMS B119) and untreated seeds were used as controls. For treatments inoculated with biological products, a dose of 100 mL/60,000 seeds was used.

The seeds were sown in 5-L pots containing sand:soil:manure (3:1:1), and the experiment was performed in a greenhouse at Londrina State University (UEL). The physical–chemical analysis of this mixture is shown in Table S1. The experiment was conducted in a completely randomized design with six repetitions. The treatments were irrigated every 2 days, and the plants were removed 45 days after sowing. The traits evaluated were (1) stem diameter, (2) plant height, (3) shoot dry mass, (4) root dry mass, and (5) shoot phosphorus content. To determine the shoot P content, the samples were dried in an oven at 70 °C for 72 h and ground in a Willey MA340 knife mill (Piracicaba, São Paulo, Brazil). Then, 0.1 g aliquots were digested in nitroperchloric solution (HNO_3_:HClO_4_) according to Malavolta et al.^38^. The P content was determined by the molybdenum blue spectrophotometric method^39^, and the readings were performed in an Agilent 8453 spectrophotometer (Agilent Technologies, California, USA) at a wavelength of 660 nm.

### Experiment under field conditions

For the tests under field conditions, maize P3340VYHR (Corteva^®^) and soybean Credenz Result I2X (BASF^®^) were used. The seeds were treated with the biological products Ag75 and Biomaphos^®^ in plastic bags using a dose of 100 mL/60,000 seeds for maize and 100 mL/50 kg seeds for soybean. Uninoculated seeds and three doses of P in the soil (25 kg P_2_O_5_, 42 kg P_2_O_5_, and 84 kg P_2_O_5_) were used as controls.

For the maize experiments, six trials were carried out: (1) Londrina (2020/2021 harvest), (2) Maringá (2020/2021 harvest), (3) Guarapuava (2020/2021 harvest), (4) Londrina (2021 harvest), (5) Londrina (2021/2022 harvest), and (6) Guarapuava (2021/2022 harvest). For soybean, five trials were conducted: (1) Londrina (2020/2021 harvest), (2) Maringá (2020/2021 harvest), (3) Guarapuava (2020/2021 harvest), (4) Londrina (2021/2022 harvest), and (5) Guarapuava (2021/2022 harvest). The physical–chemical analyses of the soils and other characteristics related to the evaluation sites are presented in Table S2.

The experiment was conducted using the complete randomized block design with four repetitions. The plots consisted of eight 6-m rows spaced 0.45 m apart with five plants per meter. Before setting up the experiment, the areas of the maize experiments were fertilized with 25 kg P_2_O_5_ ha^−1^, 60 kg K_2_O ha^−1^, and 21 kg N ha^−1^, while the soybean areas were fertilized with 25 kg P_2_O_5_ ha^−1^ and 60 kg K_2_O ha^−1^. The amounts of P_2_O_5_ applied in the experiments were considered 30% of the standard phosphate fertilization recommended for these crops. Topdressing fertilization in maize was carried out with 120 kg N ha^−1^ applied at the V6 development stage.

Five representative plants from each experimental plot were collected at the physiological maturation stage. To determine the P content in grains (maize and soybeans) and shoots (maize), the same methodology described in the previous section was used.

Grain yield (kg ha^−1^) was obtained after harvesting plants from the six central rows of each plot. The components of P use efficiency (PUsE) were determined according to Moll et al. for maize, while for soybean, grain P content was determined. P uptake efficiency (PUpE, in g of absorbed P per g of applied P) was calculated by the ratio between total plant P and P available to the plant ^40^. P utilization efficiency (PUtE, in g of grains produced per g of total P in the plant) was determined by the ratio between the grain dry biomass and the amount of total P in the plant. PUsE (in g of grains produced per g of applied P) was calculated by the product of PUpE and PUtE.

### Antagonism assay and metabolite evaluation

The Ag75 strain was activated in LBA (Acumedia, USA) at 28 °C for 24 h. For the antagonism test, this strain was inoculated on the edge of the plate containing PDA medium using a bacteriological loop. Then, a 6-mm diameter mycelial disc of the phytopathogenic fungus (*Rhizoctonia solani*, *Macrophomina phaseolina*, and *Fusarium solani*) was inoculated in the center of the plate. Then, the plates were incubated at 25 °C with a 12/12 h photoperiod. Plates with only the phytopathogenic fungus were used as controls. After 3 days for *R. solani* or 7 days for the fungi *M. phaseolina* and *F. solani*, according to the growth rate of each phytopathogenic fungus, mycelial growth in millimeters was evaluated. The percent mycelial growth inhibition was calculated using the following formula:

$$MGI\left(\%\right)=\left[\frac{dc-dt}{dc}\right]x100$$

where ‘MGI’ represents the percentage of mycelial growth inhibition, ‘dc’ is the mean colony diameter in the control and ‘dt’ is the mean colony diameter for each treatment, all measured in mm^41^.

For cell-free supernatant activity, pure colonies were suspended in saline solution (0.85% sodium chloride) and adjusted to 0.5 on the McFarland scale. To prepare the inoculum, 30 µL of this suspension was transferred to 125 mL Erlenmeyer flasks containing 30 mL of LB medium and incubated at 28 °C for 24 h at 125 rpm (Orbital shaker Nova Tecnica—NT 735, Brazil). For the production of antifungal metabolites, aliquots of 1% (v:v) of each inoculum were transferred to 1000 mL Erlenmeyer flasks containing 400 mL of AgO2 medium and incubated at 28 °C and 200 rpm in a shaker incubator in a random arrangement for 72 h. After the production step, the cultures were centrifuged at 4 °C and 9000 rpm for 10 min and filtered through 0.22 µm membranes to obtain cell-free supernatant (CFS). Subsequently, mycelial growth was evaluated in millimeters as described above. At the edge of the plate, two wells with 6 mm diameters were made equidistantly, and 200 μL of CFS was deposited in the wells. The plates were incubated at 25 °C with a 12/12 h photoperiod. As a control, plates with only the phytopathogenic fungus were used. After 3 days for *R. solani* or 7 days for the fungi *M. phaseolina* and *F. solani*, according to the growth rate of each phytopathogenic fungus, the diameter of the fungal colony was determined, and the percentage of mycelial growth inhibition was calculated.

### Genomic sequencing and gene prediction

For complete genome sequencing, Ag75 was grown in LB at 150 rpm and 28 °C for 48 h. DNA extraction was performed using a PureLinkTM Microbiome DNA Purification kit (Invitrogen, Thermo Fisher Scientific, Waltham, Massachusetts, USA). The integrity of the DNA was verified using a 1% agarose gel, and the DNA was quantified by spectrophotometry in a NanoDrop 2000/2000c (ThermoFisher Scientific, Wilmington, Delaware, USA). Sequencing was performed on the Illumina NovaSeq 6000 platform at the Institute for Cancer Research (IPEC), Guarapuava, Paraná, Brazil.

The quality of the reads and the cutoff parameters were observed and chosen using FastQC^42^. Then, with the Trimmomatic program^43^, the raw reads were filtered based on the parameters defined by FastQC. Finally, new analyses regarding the quality of the reads were performed after the filters to check if the chosen parameters were adequate.

A series of de novo assemblies were performed with different software (SPAdes and IDBA hybrid)^44^, testing diverse assembly parameters. Then, the results were compared with each other with the QUAST program^45^. Key metrics, such as total alignment size, number of contigs, highest contig, N50 values, and gene numbers (according to the reference genome provided in QUAST), were used to choose the best assembly. Using the Contiguator webserver^46^, best-assembled contigs were aligned with the *Bacillus velezensis* NKG-1 reference genome to generate the scaffolds. Gaps were manually filled, mapping reads with Bowtie2 and filling gaps using CLC Genomics Workbench 12 GUI^47^. The genome start point was determined by comparison with a reference strain genome, assuming the dnaA gene as the first gene.

The genome of the Ag75 strain was represented circularly and compared with other reference genomes using BRIG (BLAST Ring Image Generator). To determine the species, the values of ANI (average nucleotide identity) were verified with other species of *Bacillus* spp. using OrthoANI^48^. The orthoANI matrix data and information generated by the software were exported and used to create the heatmap in the R program using the ggplot2 package. The hierarchical cluster analysis used was UPGMA.

RAST software was used to predict genes related to plant growth promotion. The identified ORFs (Open Reading Frame) were submitted to functional annotation, based on the search for similarity against the Genbank non-redundant protein (nr) database, using the Blastx algorithm, with an e-value of 1.0e^−3^, and annotation of the Gene Ontology ontology terms, using the Blast2Go v2.5.0 software^49^. The identification of possible biosynthetic gene clusters (BGCs) was performed using the antiSMASH 4.0 web server^50^, which combines different genetic databases, antimicrobial molecules, and BGCs to predict the position and possible function of the clusters^51^.

### Data analysis

The agronomic data were subjected to analysis of variance, and if the assumptions were met, they were subjected to cluster analysis of Scott–Knott mean (greenhouse trials) and Tukey mean comparison test (field trials). In addition, the greenhouse data were subjected to correlation and principal component analyses. These analyses were performed by the R program using the AgroR^52^ and FactoMineR^53^ packages.

### Experimental research and field studies on plants including the collection of plant material

The authors declare that the cultivation of plants and carrying out study in Universidade Estadual de Londrina (UEL), Universidade Estadual de Maringá (UEM) and Universidade Estadual do Centro-Oeste (UNICENTRO) complies with all relevant institutional, national and international guidelines and treaties.

### Statement of permissions and/or licenses for collection of plant or seed specimens

The authors declare that the seed specimens used in this study are publicly accessible seed materials and we were given explicit written permission to use them for this research.

## Supplementary Information

Supplementary Information.

## Data Availability

The datasets generated during and analyzed during the current study are available from L.S.A.G on reasonable request.
