# Supplementary material for: Bacillus velezensis strain Ag75 as a new multifunctional agent for biocontrol, phosphate solubilization and growth promotion in maize and soybean crops
Source: Sci Rep. 2022 Sep 10;12:15284. doi: 10.1038/s41598-022-19515-8 (PMC9464197; doi:10.1038/s41598-022-19515-8)
Supplement: Supplementary file 1 — Supplementary Information. [file 41598_2022_19515_MOESM1_ESM.docx]

***Bacillus velezensis* strain Ag75 as a new multifunctional agent for biocontrol, phosphate solubilization and growth promotion in maize and soybean crops**

Mirela Mosela^1^, Galdino Andrade^1^, Luana Rainieri Massucato^2^, Suelen Regina de Araújo Almeida^2^, Alison Fernando Nogueira^2^, Renato Barros de Lima Filho^3^, Douglas Mariani Zeffa^3^, Silas Mian^2^, Allan Yukio Higashi^2^, Gabriel Danilo Shimizu^2^, Gustavo Manoel Teixeira^1^, kelvin Shinohata Branco^2^, Marcos Ventura Faria^4^, Renata Mussoi Giacomin^5^, Carlos Alberto Scapim^3^ and Leandro Simões Azeredo Gonçalves^2,3*^

^1^Universidade Estadual de Londrina (UEL), Microbiology Department, Londrina, 86051-900, Brazil.

^2^Universidade Estadual de Londrina (UEL), Agronomy Department, Londrina, 86051-900, Brazil.

^3^Universidade Estadual de Maringá (UEM), Agronomy Department, Maringá, 87020-900, Brazil.

^4^Universidade Estadual do Centro Oeste (Unicentro), Agronomy Department, Guarapuava, 85040-167, Brazil.

^5^Universidade Estadual do Centro Oeste (Unicentro), Biology Department, Guarapuava, 85040-167, Brazil.

*leandrosag@uel.br

**Supporting information**

Additional supporting information may be found in the online version of this article.

**Supplementary Table S1**. Characterization of the soil used in the greenhouse experiment.

| Characteristics^1/^ | Londrina – Greenhouse |
| --- | --- |
| Soil | Dystroferric Red Latosol |
| pH (H_2_0) | 5.50 |
| H+Al (cmolc dm^3^) | 3.24 |
| K (cmolc dm^3^) | 0.83 |
| Ca (cmolc dm^3^) | 5.81 |
| Mg (cmolc dm^3^) | 2.50 |
| Al (cmolc dm^3^) | 0.00 |
| P (mg dm^3^) | 13.30 |
| Organic matter (%) | 9.51 |

**Supplementary Table S2**. Characterization of environments used in maize and soybean experiments.

| Characteristics^1/^ | Londrina  (2020/2021) | Maringá  (2020/2021) | Guarapuava (2020/2021) | Londrina  (2021/2021) | Londrina (2021/2022) | Guarapuava  (2021/2022) |
| --- | --- | --- | --- | --- | --- | --- |
| Geographical coordinates | 23° 17’ S; 51° 10’ W | 23° 11' S; 52° 03'  W | 25° 23' S; 51º 29' W | 23° 17’ S; 51° 10’ W | 23° 17’ S; 51° 10’ W | 25° 23' S; 51º 29' W |
| Altitude (m) | 550 | 555 | 1026 | 550 | 550 | 1026 |
| Climate^2/^ | Cfa | Cfa | Cfb | Cfa | Cfa | Cfb |
| Soil | Dystroferric Red Latosol | Dystroferric Red Latosol | Dystroferric Bruno Latosol | Dystroferric Red Latosol | Dystroferric Red Latosol | Dystroferric Bruno Latosol |
| pH (CaCl_2_) | 5,2 | 5,2 | 4,5 | 5,3 | 5,4 | 5,1 |
| H+Al (cmolc dm^3^) | 3,3 | 3,2 | 5,5 | 3.5 | 3,2 | 3,9 |
| K (cmolc dm^3^) | 0,8 | 0,5 | 0,3 | 1,2 | 0,9 | 0,9 |
| Ca (cmolc dm^3^) | 5,4 | 2,9 | 2,1 | 5,8 | 5,4 | 2,7 |
| Mg (cmolc dm^3^) | 1,6 | 1,3 | 0,6 | 1,2 | 1,5 | 1,2 |
| Al (cmolc dm^3^) | 0,1 | 0,0 | 0,9 | 0,1 | 0,0 | 0,5 |
| P (mg dm^3^) | 22,6 | 10,7 | 7,0 | 12,3 | 13,4 | 9,0 |
| Organic Matter (%) | 2,8 | 1,8 | 4,2 | 2,5 | 2,6 | 4,4 |

^1/^ Physical-chemical analyses were performed using soil layer samples from 0 to 20 cm.

^2/^ Köppen climate classification = Cfa, Humid subtropical climate; Cfb: Temperate oceanic climate.

**Supplementary Table S3**. Analysis of variance for five agronomical traits evaluated in maize in greenhouse with seeds inoculated with different phosphate-solubilizing bacteria.

| Source of  Variation | DF | Mean Square^1/^ | | | | |
| --- | --- | --- | --- | --- | --- | --- |
|  |  | SD | PH | RDM | SDM | SPC |
| Treatments | 14 | 6.72^**^ | 354.98^**^ | 1.34^**^ | 6.41^**^ | 1.95^**^ |
| Error | 75 | 2.16 | 82.08 | 0.32 | 1.63 | 0.26 |
| Mean |  | 11.90 | 74.2 | 1.85 | 6.58 | 4.43 |
| CV (%) |  | 12.37 | 12.21 | 30.49 | 19.39 | 11.63 |

^1^SD: stem diameter, PH: plant height, RDM: root dry mass, SDM: shoot dry mass, and SPC: shoot phosphorus content.

*ns*, ** e * indicates non-significance, significance at levels 1 and 5% of probability by the F test, respectively

**Supplementary Table S4**. Mean clustering test (Scott-Knott) for stem diameter (SD), plant height (PH), root dry mass (RDM), shoot dry mass (SDM) and shoot phosphorus content (SPC) in maize seeds inoculated with phosphorus solubilizing bacteria in a greenhouse experiment.

| Treatments | Traits | | | | |
| --- | --- | --- | --- | --- | --- |
|  | SD | PH | RDM | SDM | SPC |
| Control | 12.18 **a** | 72.83 **b** | 1.85 **c** | 6.16 **b** | 3.99 **b** |
| Biomaphos | 13.15 **a** | 71.17 **b** | 2.11 **b** | 6.83 **a** | 4.43 **b** |
| Strain01 | 11.27 **b** | 70.83 **b** | 1.65 **c** | 6.25 **b** | 3.79 **b** |
| Strain02 | 13.13 **a** | 76.67 **a** | 2.04 **b** | 7.53 **a** | 4.40 **b** |
| Strain03 | 13.67 **a** | 84.67 **a** | 2.68 **a** | 8.78 **a** | 5.39 **a** |
| Strain04 | 12.95 **a** | 80.33 **a** | 2.79 **a** | 7.72 **a** | 4.66 **a** |
| Strain05 | 11.62 **b** | 67.33 **b** | 1.25 **c** | 6.03 **b** | 5.06 **a** |
| Strain06 | 10.88 **b** | 74.17 **b** | 1.47 **c** | 6.33 **b** | 4.28 **b** |
| Strain07 | 9.60 **b** | 58.33 **c** | 1.14 **c** | 4.74 **b** | 5.12 **a** |
| Strain08 | 11.56 **b** | 61.83 **c** | 1.54 **c** | 5.43 **b** | 4.70 **a** |
| Strain09 | 11.37 **b** | 71.50 **b** | 1.98 **b** | 6.67 **b** | 4.40 **b** |
| Strain10 | 11.80 **b** | 79.50 **a** | 2.03 **b** | 6.37 **b** | 4.20 **b** |
| Strain11 | 12.15 **a** | 80.50 **a** | 2.10 **b** | 7.29 **a** | 3.85 **b** |
| Strain12 | 10.95 **b** | 79.00 **a** | 1.67 **c** | 5.90 **b** | 4.17 **b** |
| Strain13 | 12.24 **a** | 84.33 **a** | 1.83 **c** | 7.34 **a** | 4.78 **a** |

^1/^ Means followed by the same letter in the column do not differ statistically at 5% probability by the Scott-Knott test.

**Supplementary Table S5**. Effects of phosphorus solubilizing bacteria on grain yield in maize in six experiments.

| Treatments | Experiments^1/^ | | | | | |
| --- | --- | --- | --- | --- | --- | --- |
|  | Env.1 | Env.2 | Env.3 | Env.4 | Env.5 | Env.6 |
| Control 25 Kg P_2_O_5_ | 8291 **b** | 6475 **b** | 4679 **b** | 4486 **a** | 8317 **b** | 6778 **b** |
| Control 42 Kg P_2_O_5_ | 9108 **ab** | 6555 **b** | 5481 **ab** | 5176 **a** | 9978 **a** | 7264 **ab** |
| Control 84 Kg P_2_O_5_ | 9477 **a** | 7822 **a** | 6088 **a** | 5322 **a** | 10856 **a** | 7539 **a** |
| 25 Kg P_2_O_5_ + Biomaphos | 8608 **ab** | 6842 **ab** | 5407 **ab** | 4953 **a** | 10147 **a** | 7294 **ab** |
| 25 Kg P_2_O_5_ + Ag75 | 9210 **ab** | 7369 **ab** | 5829 **ab** | 5043 **a** | 11206 **a** | 7306 **ab** |

^1/^Env1.: Londrina (2020/2021), Env2.: Maringá (2020/2021), Env3.: Guarapuava (2020/2021), Env4.: Londrina (2021/2021), Env5.: Londrina (2021/2022) and Env6.: Guarapuava (2021/2022).

**Supplementary Table S6**. Effects of phosphorus solubilizing bacteria on grain yield in soybean in five experiments.

| Treatments | Experiments^1/^ | | | | |
| --- | --- | --- | --- | --- | --- |
|  | Env.1 | Env.2 | Env.3 | Env.4 | Env.5 |
| Control 25 Kg P_2_O_5_ | 2058 **b** | 2052 **b** | 3580 **b** | 3195 **b** | 2936 **b** |
| Control 42 Kg P_2_O_5_ | 2512 **ab** | 2708 **ab** | 3899 **ab** | 3374 **ab** | 3523 **ab** |
| Control 84 Kg P_2_O_5_ | 2945 **a** | 2780 **a** | 4313 **a** | 4002 **a** | 3449 **ab** |
| 25 Kg P_2_O_5_ + Biomaphos | 2240 **ab** | 2344 **ab** | 4494 **a** | 3389 **ab** | 3754 **a** |
| 25 Kg P_2_O_5_ + Ag75 | 2843 **a** | 2694 **ab** | 4013 **ab** | 3808 **ab** | 3334 **ab** |

^1/^Env1.: Londrina (2020/2021), Env2.: Maringá (2020/2021), Env3.: Guarapuava (2020/2021), Env4.: Londrina (2021/2022) and Env5.: Guarapuava (2021/2022).

**Supplementary Table S7.** Biosynthetic Gene Clusters (BGCs) found within *Bacillus velezensis* Ag109 genome using the webserver antiSMASH 5.1.0.

| Cluster | Type | From (pb) | To (pb) | Most similar known cluster | | Similarity (%) |
| --- | --- | --- | --- | --- | --- | --- |
| 1 | thiopeptide | 281.447 | 311.183 | - | - | - |
| 2 | NRPS | 322.005 | 387.087 | Surfactin | NRP:Lipopeptide | 82 |
| 3 | PKS-like | 918.587 | 959.831 | Butirosin A and B | Saccharide | 7 |
| 4 | Terpene | 1045.457 | 1062.628 |  |  |  |
| 5 | TransAT-PKS | 1392.865 | 1479.251 | Macrolactin | Polyketide | 100 |
| 6 | TransAT-PKS, T3PKS, NRPS | 1705.384 | 1805.935 | Bacilaene | Polyketide + NRP | 100 |
| 7 | NRPS, TransAT-PKS, Betalactone | 1874.238 | 2011.614 | fengycin | NRP | 100 |
| 8 | Terpene | 2034.651 | 2056.534 | - | - | - |
| 9 | T3PKS | 2140.205 | 2181.305 | - | - | - |
| 10 | TransAT-PKS | 2313.205 | 2406.997 | Difficidin | Polyketide + NRP | 100 |
| 11 | NRPS, RiPP-like | 3039.587 | 3091.378 | Bacillibactin | NRP | 100 |
| 12 | Other | 3619.927 | 3661.345 | Bacilysin | Other | 100 |

NRPS, non-ribosomal peptide synthetase; NRP, non-ribosomal peptide. PKS, polyketide synthetase. AT, acetyltransferase; T3PKS, type 3 Pks.

**
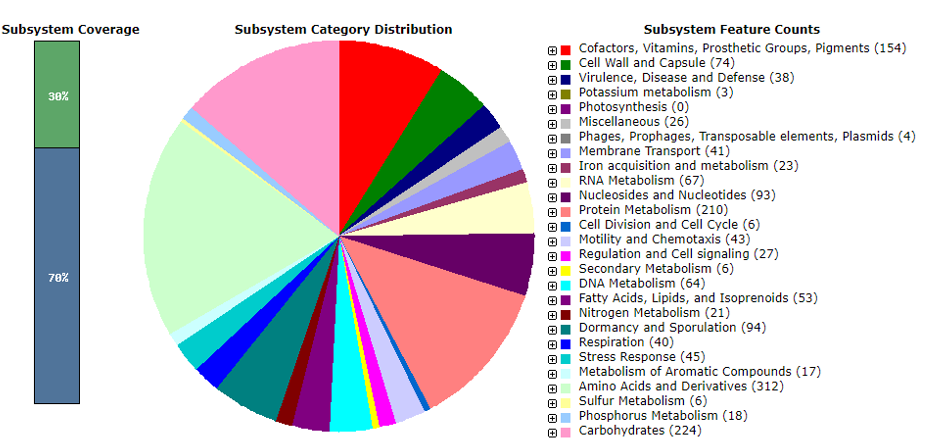
**

**Supplementary Figure S1.** SEED classification of Ag75 genome. Pie chart depicting functional categories in Ag75 genome. The SEED annotated genome was compared to hundreds of genomes maintained within the SEED integration. RAST annotation (server possessed identified protein encoding genes (PEGs), RNA genes and repeat regions) was used to create the pie chart.
